# The Glioma-IRE project − Molecular profiling in patients with glioma: steps toward an individualized diagnostic and therapeutic approach

**DOI:** 10.1186/s12967-023-04057-y

**Published:** 2023-03-23

**Authors:** Veronica Villani, Beatrice Casini, Antonio Tanzilli, Mario Lecce, Fabrizio Rasile, Stefano Telera, Andrea Pace, Francesca Piludu, Irene Terrenato, Francesca Rollo, Francesca De Nicola, Maurizio Fanciulli, Matteo Pallocca, Gennaro Ciliberto, Mariantonia Carosi

**Affiliations:** 1 Neuro-Oncology Unit, IRCCS Regina Elena National Cancer Institute, Via Elio Chianesi 53, 00144 Rome, Italy; 2 Pathology Unit, IRCCS Regina Elena National Cancer Institute, Via Elio Chianesi 53, 00144 Rome, Italy; 3 Division of Neurosurgery, IRCCS Regina Elena National Cancer Institute, Via Elio Chianesi 53, 00144 Rome, Italy; 4 Radiology and Diagnostic Imaging Department, IRCCS Regina Elena National Cancer Institute, Via Elio Chianesi 53, 00144 Rome, Italy; 5 UOSD Clinical Trial Center Biostatistics and Bioinformatics, IRCCS Regina Elena National Cancer Institute, Via Elio Chianesi 53, 00144 Rome, Italy; 6 Department of Research, Diagnosis and Innovative Technologies, Translational Research Area, IRCCS Regina Elena National Cancer Institute, Via Elio Chianesi 53, 00144 Rome, Italy; 7 Scientific Direction, IRCCS National Cancer Institute Regina Elena, Via Elio Chianesi 53, 00144 Rome, Italy

**Keywords:** Glioma, Glioblastoma, Next-generation sequencing, Molecular profiling, Precision medicine

## Abstract

**Background:**

This study aimed to characterize the genetic profile of patients with glioma and discuss the impact of next-generation sequencing in glioma diagnosis and treatment.

**Methods:**

Between 2019 and 2022, we analyzed the genetic profile of 99 patients with glioma through the Oncomine Focus Assay. The assay enables the detection of mutations in 52 driver genes, including single nucleotide variants (SNVs), copy number variants (CNVs), and gene fusions. We also collected and analyzed patients’ clinic characteristics and treatment outcomes.

**Results:**

Over a period of 35 months, 700 patients with glioma followed by our neuro-oncology unit were screened, and 99 were enrolled in the study; most of the patients were excluded for inadequate non-morphological MRI or lack/inadequacy of the tissue samples. Based on our findings, most patients with glioma present mutations, such as SNVs, CNVs or gene fusions. Our data were similar to those reported by The Cancer Genome Atlas Program in terms of frequency of SNVs and CNVs, while we observed more cases of gene fusions. Median overall survival, progression-free survival, and time to progression were significantly lower for patients with grade VI glioblastoma than those with other gliomas. Only four patients were offered a targeted treatment based on the mutation detected; however, only one received treatment, the others could not receive the selected treatment because of worsening clinical status.

**Conclusion:**

Routine timely molecular profiling in patients with glioma should be implemented to offer patients an individualized diagnostic approach and provide them with advanced targeted therapy options if available.

## Background

Gliomas are the most common primary tumors of the central nervous system (CNS), with an estimated annual incidence of six to seven cases per 100,000 individuals and a median age of onset around 50–60 years of age [1, 2]. Despite multimodal approaches and different therapeutic strategies, these tumors still pose a significant challenge to healthcare professionals, and patient prognosis is often poor [3]. Therapeutic options are especially limited in the case of grade VI glioblastoma (GBM), one of the most frequent types of primary brain tumors, accounting for 60% of all CNS tumors and for which median survival is only 15–20 months [4].

Gliomas comprise a genetically, histologically, and clinically heterogeneous group of tumors, which are classified based on their presumed cell of origin (astrocytomas, oligodendrogliomas, ependymomas, or mixed gliomas) and the level of malignancy or grade, which is determined by tumor cell density, mitotic index and presence of nuclear atypia and necrosis [5]. This microscopic-based classification, which has represented the cornerstone for glioma diagnosis and management for decades, has proven to be rather limiting, as histologically identical tumors may present very different clinical features, natural history, and response to treatment [3]. For this reason, during the last decade, the focus has progressively shifted from general histopathological characterization toward a deeper understanding of gliomas’ molecular and genetic alterations. Starting from the 2016 WHO classification of CNS, traditional histological criteria have been supplemented with genomic biomarkers [6]. Recently, the new WHO classification is based only on molecular profile [7].

The advent of high-throughput technologies for molecular testing, such as microarray-based procedures and next-generation sequencing (NGS), has expanded the capacity for large-scale mutations analysis, allowing the identification of multiple novel diagnostic, prognostic and/or predictive biomarkers [8–11].

Some of the most frequent mutations identified in gliomas include the isocitrate dehydrogenase (*IDH*) 1 or 2 gene mutation (*IDH* mutation), which is associated with a survival benefit in patients with high-grade glioma, [12]; the codeletion of chromosomal arms 1p and 19q (1p/19q codeletion), which is both a diagnostic marker for oligodendroglial CNS and a strong predictor of chemotherapeutic and radiotherapeutic response [13]; the loss of nuclear a-thalassemia/mental retardation X-linked syndrome, which is a hallmark of astrocytic tumors [14]; and O6-methylguanine DNA methyltransferase promoter methylation, which is an independent predictor of survival and response to combined radiotherapy and temozolomide [8, 15]. Large-scale molecular profiling studies have also identified different genetic and epigenetic aberration profiles that may be employed for tumor classification, such as mutations in B-Raf proto-oncogene, serine/threonine kinase (*BRAF*) gene, cyclin-dependent kinase inhibitor 2A (*CDKN2A*) gene, tumor protein p53 (*TP53*), phosphatase and tensin homolog (*PTEN*) gene, telomerase reverse transcriptase (*TERT*) promoter, as well as amplifications of proto-oncogenes, such as epidermal growth factor receptor (*EGFR*) [16].

A deeper understanding of the role of these and other molecules in glioma pathogenesis is the first step for a more precise diagnosis and for developing new targeted therapies that could improve the current patient prognosis. Currently, the standard of care for newly diagnosed high-grade glioma is limited to surgical resection followed by adjuvant chemotherapy (temozolomide) and radiotherapy. However, clinical response is still poor, and almost all patients with GBM experience tumor progression [17, 18].

In this paper, we present the results of molecular genetic profiling of 99 patients with glioma. We discuss the insight on glioma management derived from our findings, as well as the role of NGS-based molecular sequencing in glioma diagnosis and treatment, and the future perspectives on the implementation of precision medicine for glioma.

## Methods

### Study design

This was a monocentric prospective observational study conducted at the IRCSS National Cancer Institute Regina Elena (Italy) between 1 December 2019 and October 2022. The study aimed to characterize the molecular profile of patients with glioma, to better understand whether there is a correlation between non-morphological data on brain MRI obtained with diffusion and perfusion techniques with molecular data, and to implement a new model for molecular diagnostics based on NGS analysis. In this paper, we only reported data on molecular profiling, while no data on the correlation with radiological results are presented.

The study included patients with glioma at diagnosis or recurrence, with morphological and non-morphological MRI before surgery, and with sufficient tumor tissue for the analysis. Patients with not available, scarce or not adequate tumor tissue were excluded from the study.

The study was approved by the local Ethics Committee, and all the patients signed an informed consent to participate in the trial.

### Molecular analysis and data collection

Molecular analysis was conducted using an NGS approach through the Oncomine Focus Assay (Thermo Fisher Scientific). This targeted sequencing assay allows the identification of biomarkers in 52 genes with known relevance in solid tumors, enabling the detection of hotspots, single nucleotide variants (SNVs), indels, copy number variants (CNVs), and gene fusions. The assay also allows the concomitant assessment of DNA and RNA in a single workflow.

In addition, we collected data on patients’ demographic and clinical features, including previous treatments and outcomes in terms of treatment response, overall survival (OS), progression-free survival (PFS) and time to progression from the NGS analysis (TTP).

#### Tissue selection and DNA-RNA extraction

Four or five Sects. (5 µm thick) from neoplastic tissue embedded in paraffin blocks (FFPE) were cut and macro-dissected for enrichment in neoplastic tissue. DNA and RNA extraction was carried out with the DNA FFPE tissue kit and RNeasy FFPE kit Qiagen (QIage, Hilden, Germany) according to the manufacturer’s instructions and volume of elution of 20 µl. Nucleic acid concentration was measured with a fluorimetric method, Qubit 4 fluorometer (Thermo Fisher Scientific) and with the DNA 1X dsDNA HS (High Sensitivity) assay kit and RNA HS assay kit, which are particularly indicated for FFPE and highly degraded DNA/RNA samples. Only concentrations greater than 1 ng/µl were considered acceptable.

#### Library preparation

An automatic library was prepared with 0.67 ng of input DNA and the Ion AmpliSeq™ Kit for the Thermo Fisher Scientific instrument Chef System DL8 (eight samples every run) (Thermo Fisher Scientific, Waltham, MA, USA). The Ion AmpliSeq^™^ Kit for Chef DL8 includes the reagents and materials for the automated preparation of up to eight barcoded Ion AmpliSeq^™^ libraries. For each sample, the Ion Chef^™^ instrument sets up a target amplification reaction with the primer pool using a different barcode for each sample. Library concentration was normalized to approximately 100 pM using Ion Library Equalizer^™^ technology, and libraries were combined in a single tube ready to use in Ion Chef^™^ for template preparation reactions. However, a quantification of the library pool was performed with the Qubit kit 1X dsDNA HS assay; the measure was taken in ng/ml and converted in pM, assuming that 15 ng/ml corresponded to 100 pM. For RNA libraries, 10 ng of total RNA for each sample was retro-transcribed in a 96-well PCR plate using the 5X VILO^™^ Reaction Mix and 10X SuperScript™ Enzyme Mix according to manufacturer indication. The library preparation was performed with the cDNA synthetized with Ion AmpliSeq^™^ Kit for Chef DL8, as previously described.

DNA and RNA library pools were mixed in a ratio of 4:1, and then the pooled library was diluted to 33 PM.

#### Template preparation

The final pool was put in the Ion 520^™^ & Ion 530^™^ Kit—Chef^™^ with the reagents and materials required by the Ion Chef^™^ Instrument to prepare template-positive Ion Sphere™ Particles and load the sequencing support, in this case, a 520^™^ Chip. The high-throughput sequencing was performed in the Ion Gene Studio S5 Prime system (Thermo Fisher Scientific) Run planned in the S5 Torrent.

Sequences were aligned to the hg19 reference genome, and variant calling was performed using Ion Reporter version 5.4 (Thermo Fisher Scientific, Waltham, MA, USA) by automatically uploading data files from the Torrent Suite^™^ Software. The workflow analysis was Oncomine^™^ Focus—520—w2.4—DNA and Fusions—Single Sample. This workflow detects and annotates low-frequency somatic variants (SNPs, INDELs, CNVs) from targeted DNA libraries, as well as gene fusions from targeted RNA libraries, of the Oncomine™ Focus Assay. The system can display which variants are known to be cancer drivers.

The Oncomine Focus panel included the analyses of hotspot variants in the following genes: *AKT1, ALK, AR, BRAF, CDK4, CTNNB1, DDR2, EGFR, ERBB2, ERBB3, ERBB4, ESR1, FGFR2, FGFR3, GNA11, GNAQ, HRAS, IDH1, IDH2, JAK1, JAK2, JAK3, KIT, KRAS, MAP2K1, MAP2K2, MET, MTOR, NRAS, PDGFRA, PIK3CA, RAF1, RET, ROS1, SMO*. The genes evaluated for CNVs were: *ALK, AR, BRAF, CCND1, CDK4, CDK6, EGFR, ERBB2, FGFR1, FGFR2, FGFR3, FGFR4, KIT, KRAS, MET, MYC, MYCN, PDGFRA, PIK3CA*. The gene analyzed for fusion were: *ABL1, AKT3, ALK, AXL, BRAF, EGFR, ERBB2, ERG, ETV1, ETV4, ETV5, FGFR1, FGFR2, FGFR3, MET, NTRK1, NTRK2, NTRK3, PDGFRA, PPARG, RAF1, RET* and *ROS1*.

Annotation criteria for SNPs indels variants were: Allele Frequency ≥ 0.05 and Alternate Allele Observation Count ≥ 10. Annotation criteria for copy number amplification were: 5% CI value ≥ 4 when two copies are expected (diploid status). Positive fusion call was annotated if it corresponded to one of the 271 Oncomine™ Focus fusion variants or if an imbalance value of the 3' and 5' reads were detected. Filtered variants were manually reviewed in a COSMIC database for somatic variations, ClinVar of the National Center for Biotechnology Information, VarSome tool for clinical interpretation of NGS data, as well as in the scientific literature to exclude polymorphisms or non-pathogenic variants. To determine the actionability of the variant, we consulted the OncoKB MSK's Precision Oncology Knowledge Base [19] and Oncomine Reporter, a genomic analysis software tool developed by Thermo Fisher Scientific for further examination of NGS data. Classification of variants’ actionability was performed according to OncoKB [19], guidelines of the Association for Molecular Pathology, American Society of Clinical Oncology, and College of American Pathologists [20].

### Statistical analysis

Demographic variables, patient clinical features, and outcome measures were reported using descriptive statistics. Pearson’s Chi-square non-parametric test was used to compare groups. Survival curves ((OS, PFS, and TTP) were estimated with the Kaplan–Meier method and compared using the log-rank test. A p < 0.05 was considered statistically significant. All analyses were carried out with SPSS v.21.0.

## Results

Over a period of 35 months, 700 patients with glioma were screened, and 99 were enrolled in the study. Most patients were excluded for inadequate non-morphological MRI, while few patients were excluded because of lack or inadequacy of the tissue samples. Samples from 99 patients were analyzed through the NGS DNA mutation assay. Patients’ demographics and clinical characteristics are reported in Table 1.

**Table 1:** Patient’s characteristics: mutational analysis of overall sample

Characteristics	n = 99, n (%)
Gender	
• Male	65 (66)
• Female	34 (34)
• Age at diagnosis (years), median (min–max)	57 (19–81)
• Median (min–max)	57 (19–81)
Histology	
• GBM	66 (67)
• Other glioma	33 (33)
*MGMT* (missing)	
• Methylated	39 (42)
• Not methylated	53 (58)
Type of tissue	
• Diagnosis	73 (74)
• Relapse	26 (26)
Previous treatments (CT and or RT)	
• Yes	25 (25)
• No	73 (75)
Clinical response at NGS panel determination (7 not evaluable)	
• CR/PR/NED	13 (14)
• SD	15 (16)
• PD	65 (70)
OS status (at 35 months)	
• Alive	45 (45)
• Dead	54 (55)

*CR* complete response, *CT* chemotherapy, *GBM* glioblastoma multiforme, *MGMT* O6-methylguanine DNA methyl-transferase, *NED* no evidence of disease, *NGS* next-generation sequencing, *OS* overall survival, *PD* progressive disease, *PR* partial response, *RT* radiotherapy, *SD* stable disease

Most of the patients were male (66% vs 34%), the median age at diagnosis was 57 years old (range 19–81), and the majority of the patients (67%) had a diagnosis of GBM. Only 25% of the patients had received previous chemotherapy or radiotherapy; most of the patients progressed after treatment (70%), 16% reached a stable disease, and 14% achieved a complete response/partial response or no evidence of disease (Table 1).

### NGS results

**Fig. 1:**
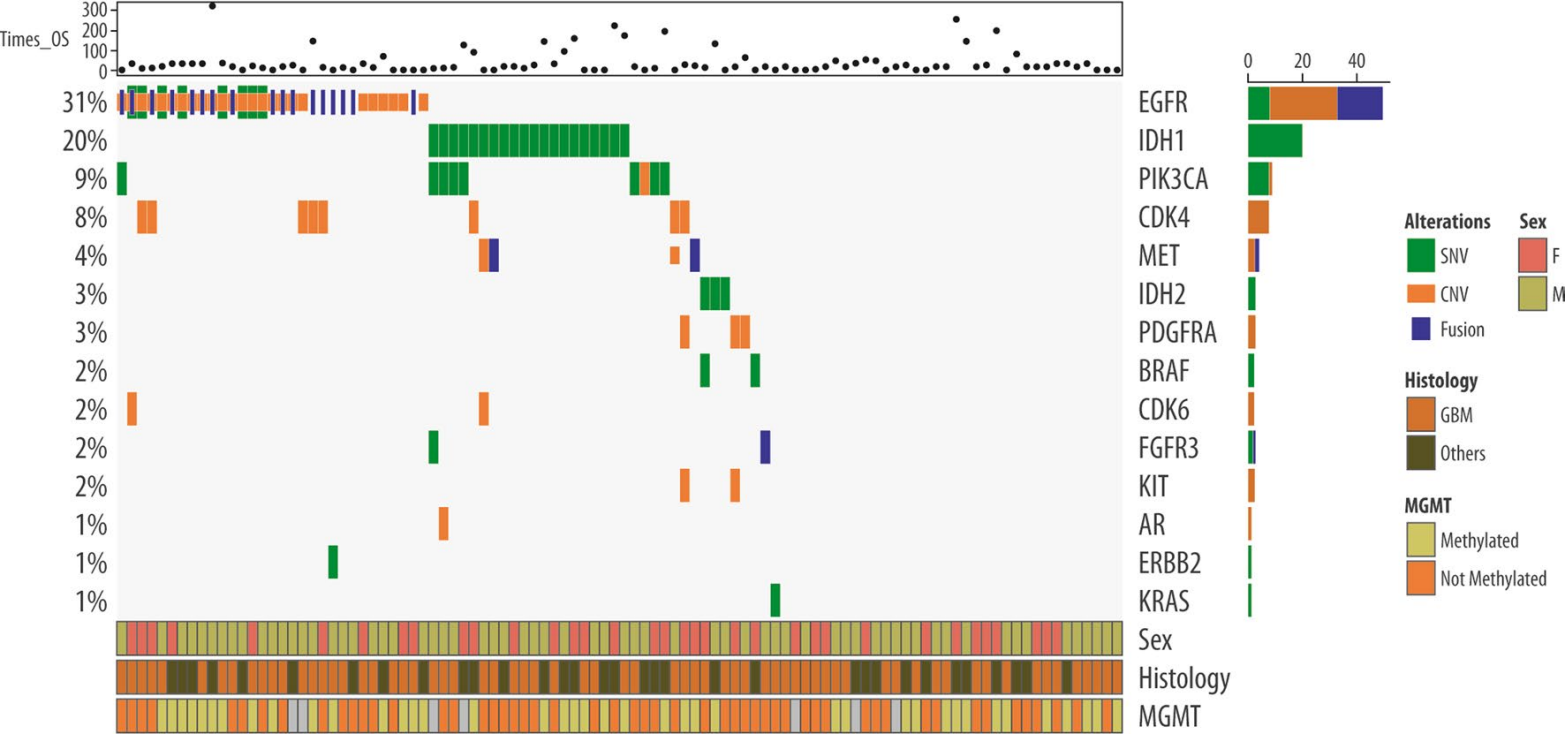
Results of NGS

The results of mutational profiling are reported in Table 2 and in Fig. 1. Overall, 67 patients out of 99 had at least one mutation (SNV, CNV or fusion).

**Table 2:** Mutational Results: SNVs, CNVs and Fusions

	Our sample, n (%)	TCGA-Public DB*, n (%)	Treatments options
SNV	n = 99	n = 2861	
SNV total			
• Mutated	38 (38)	1403 (49)	
• WT	61 (62)	1458 (51)	
*IDH1*:			
• Mutated	20 (20)	924 (32)	TIER IIC
• WT	79 (80)	1937 (68)	
*IDH2*			
• Mutated	3 (3)	45 (2)	TIER IIC
• WT	96 (97)	2816 (98)	
*EGFR*			
• Mutated	8 (8)	341 (12)	TIER IIC
• WT	91 (92)	2520 (88)	
*BRAF*			
• Mutated	2 (2)	71 (2)	TIER IA, TIER IIC
• WT	97 (98)	2790 (98)	
*PIK3CA*			
• Mutated	8 (8)	236 (8)	TIER IIC
• WT	91 (92)	2625 (92)	
*FGFR3*			
• Mutated	1 (1)	33 (1)	TIER IIC
• WT	98 (99)	2828 (99)	
*ERBB2*			
• Mutated	1 (1)	24 (1)	TIER IIC
• WTWT	98 (99)	2837 (99)	
*KRAS*			
• Mutated	1 (1)	25 (1)	Basket trials of all tumors
• WT	98 (99)	2836 (99)	
CNV	n = 99	n = 2861	
CNV total			
• Amplified	35 (35)	770 (27)	
• WT	64 (65)	2091 (73)	
*EGFR*			
• Amplified	25 (25)	519 (18)	TIER IIC
• WT	74 (75)	2342 (82)	
*PIK3CA*			
• Amplified	1 (1)	23 (1)	TIER IIC
• WT	98 (99)	2838 (99)	
*MET*			
• Amplified	2 (2)	40 (1)	TIER IIC
• WT	97 (98)	2821 (99)	
*CDK4*			
• Amplified	8 (8)	201 (7)	TIER IIC
• WT	91 (92)	2660 (93)	
*CDK6*			
• Amplified	2 (2)	36 (1)	TIER IIC
• WT	97 (98)	2825 (99)	
*PDGFRA*			
• Amplified	3 (3)	156 (6)	
• WT	96 (97)	2702 (94)	
*KIT*			
• Amplified	2 (2)	116 (4)	
• WT	97 (98)	2745 (96)	
Fusion	n = 99	n = 514**	
Fusion total			
• Yes	21 (21)	11 (2)	
• No	78 (79)	503 (98)	
*EGFR*			
• Yes	17 (17)	5 (1)	TIER II C
• No	82 (83)	509 (99)	
*RET*			
• Yes	1 (1)	0	TIER IA all tumours
• No	98 (99)	514	
*MET*			
• Yes	2 (2)	2 (1)	TIER II C
• No	97 (98)	512 (99)	
*FGFR3*			
• Yes	1 (2)	4 (1)	TIER II C
• No	98 898)	510 (99)	

*CNV* copy number variants, *DB* database, *GBM* glioblastoma multiforme, *LGG* low-grade glioma, *SNV* single nucleotide variants, *TCGA* The Cancer Genome Atlas Program, *WT* wild-type

TIER: Evidence-based variant categorization into four tiers [20]

^*^TCGA Pan Cancer Atlas + GLASS Consortium (Nature 2019) + MSK (Clin Cancer Researc2019) + TCGA Firehose Legacy [20–33]

^**^Only LGG

Comparing the frequency of SNVs according to tumor histology, we found that they were significantly less frequent in GBM patients (31.6%; p < 0.001) than in other glioma patients (68.4% of all SNVs; data not shown). In detail, the *IDH1* variant was more present in other gliomas than in GBM (p < 0.001). Other variants, such as *IDH2, BRAF,* and *PIK3A,* had low-frequency and were mainly present in patients with other gliomas, while low-frequency *EGFR* variants were more common in GBM patients.

As shown in Table 2, 38 patients had at least one SNV: in detail, 33 patients had only one SNV, four patients had two SNVs, and one patient had three SNVs. The most commonly mutated gene was *IDH1* (20%), followed by *PIK3CA* (8%) and *EGFR* (8%); all the other genes (*IDH2, BRAF, FGFR3, ERBB2* and *KRAS*) had variable mutation rates (1–3%). Patients with multiple variants presented *IDH1* + *PIK3CA* or *BRAF* mutation, while the only patient with three variants had a combination of *IDH1* + *PIK3CA* + *FGF3* mutations. Comparing our data with those reported in the dataset of The Cancer Genome Atlas Program (TCGA) [21–34], we observed a similar frequency of SNV for *IDH2, EGFR*, *BRAF, PIK3A, FGFR3, ERBB2* and *KRAS*; conversely, we detected a lower frequency of *IDH1* in our database compared with TCGA.

The frequency of CNVs was higher in GBM (82.9% of all CNVs) than in other gliomas (17.1%; p = 0.011). In particular, *EGFR* (22/26) and *CDK4* (6/8) mutations were more present in patients with GBM than those with other gliomas, whereas PDGFRA CNVs were present only in patients with GBM (3/3; data not shown).

As shown in Table 2, 35 patients had at least one CNV: in detail, 26 patients had only one CNV, seven patients had two CNVs, and one patient had three CNVs. The most commonly mutated gene in terms of copy number was *EGFR* (25%), followed by *CDK4* (8%); all the other genes (*PDGFRA, MET, CDK6, KIT* and *PIK3CA*) were mutated in approximately 1–3% of the cases. Patients with multiple CNVs mostly presented mutations in the genes *EGFR* + *CDK4/6* (four patients out of seven). These results are similar to data reported in the TCGA database.

As shown in Table 2, 21 patients had at least one gene fusion; in most cases, gene fusions involved the *EGFR* gene (17%), followed by *MET* (2%), *FGFR3* (2%), and *RET* (1%). Fusions were more present in GBM (90.5% of all fusion cases) than in other gliomas (9.5%; p = 0.009 F-fisher), and in particular, *EGFR* fusion was present in 16/17 of the patients with GBM. When we compared our data with those reported in the TCGA, we observed a higher frequency of fusions in our dataset. This result is probably because the TCGA database on gene fusions refers only to patients with low-grade glioma; therefore, data are not entirely comparable.

### Clinical outcomes and actionable mutations

**Fig. 2:**
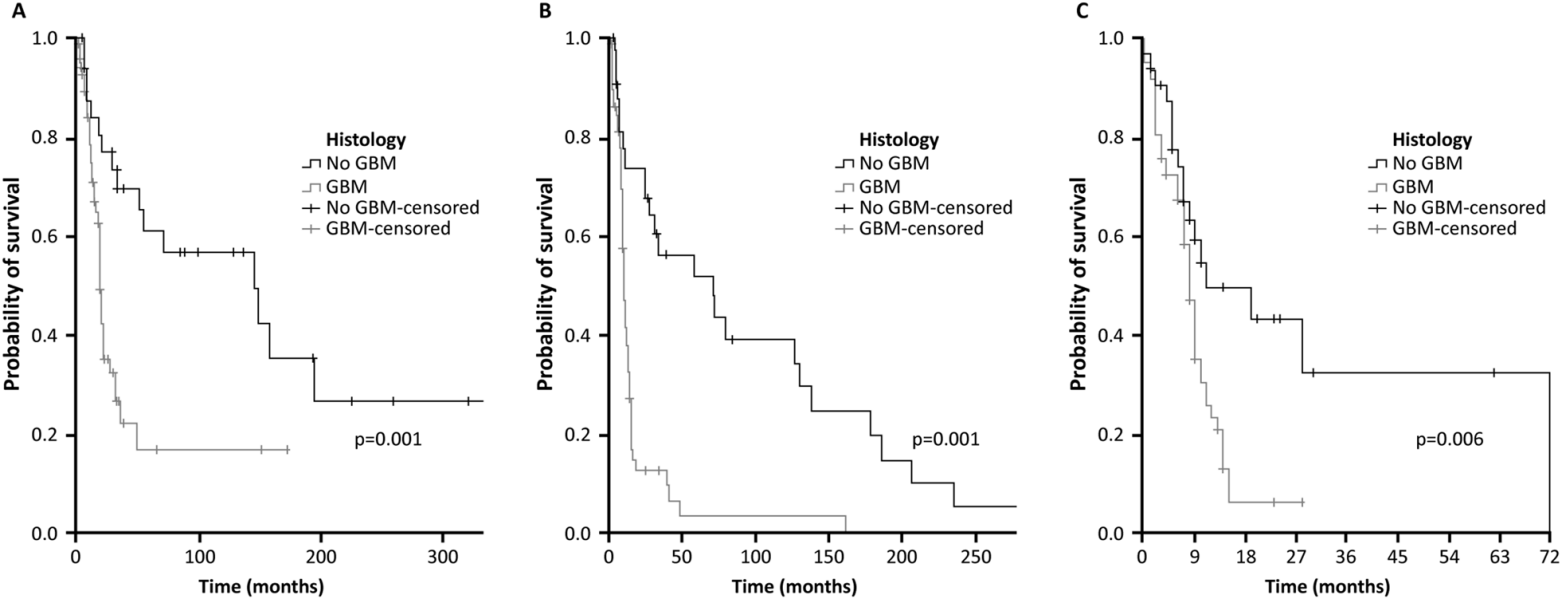
Survival parameters in patients with GBM vs no GBM histology: overall survival (**A**), progression-free survival (**B**) and time to disease progression (**C**)

Median OS was 27 months (range 14.9–39.1), with significantly superior results for patients with no GBM histology (145; 26.8–263.2) than for those with GBM (19; 17.3–20.8; p = 0.001) (Fig. 2A). Median PFS was 12 months (9.0–15.0), with a significant difference between patients with no GBM histology (71; 15.5–126.5) compared with those with GBM (10; 8.4–11.6; p < 0.001) (Fig. 2B). Lastly, TTP after NGS was 9 months overall (7.8–10.2) with significantly superior results for no GBM (11; 0–23.2) vs GBM (8; 6.9–9.1; p = 0.006) (Fig. 2C).

**Fig. 3:**
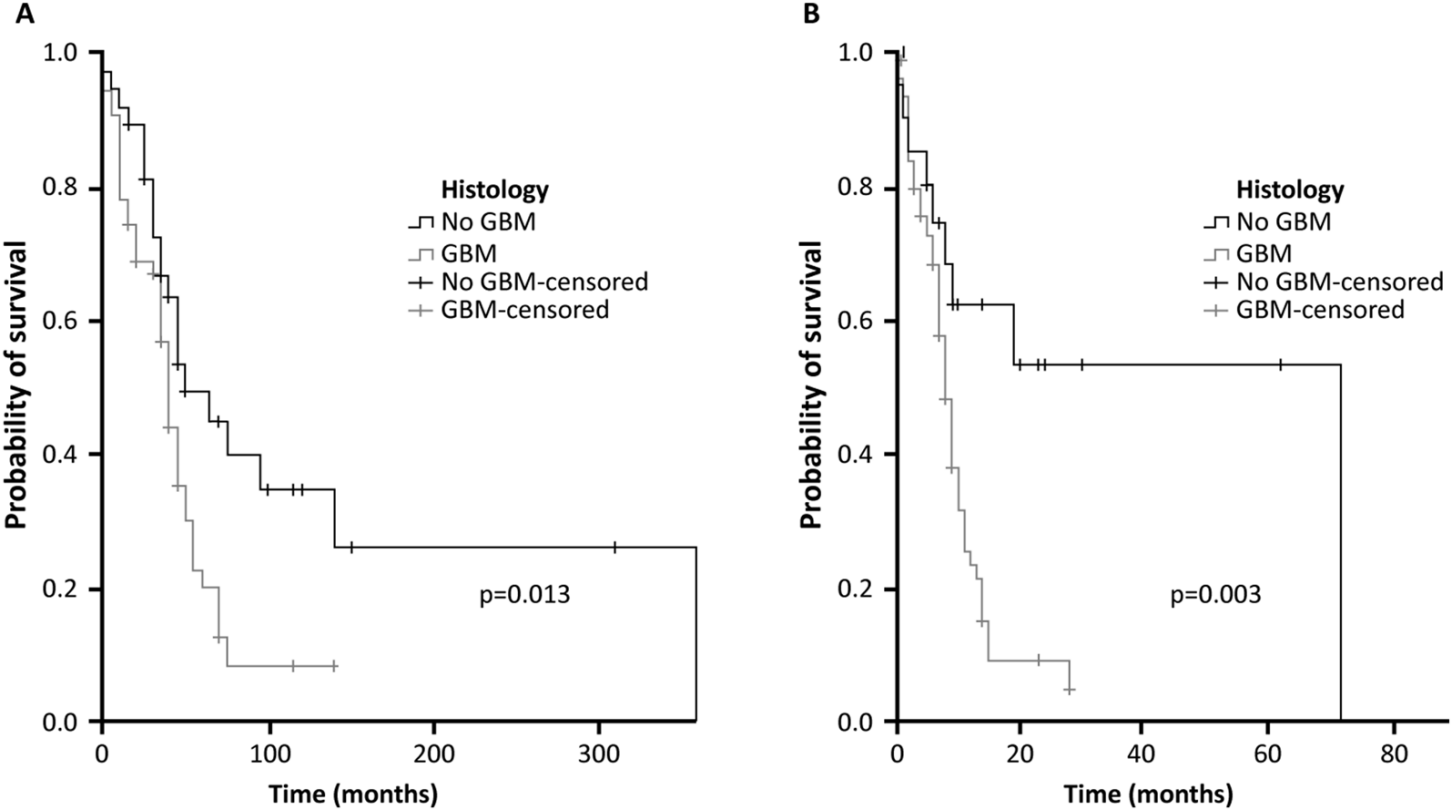
Impact of molecular profile on glioma outcome: Time to disease progression in patients with at least one SNV vs no SNV (**A**) and in patients with *IDH1* SNV vs *IDH1* WT (**B**)

Clinical response was better in patients with at least one SNV than in those without SNV [TTP 8 months (range 6.9–9.1) vs 10 months (5.3–14.7); p = 0.013] (Fig. 3A). In particular, variations in *IDH1* were associated with significantly improved TTP than *IDH1 WT* (8 months [range 6.8–9.2] vs 72 months [range not reached yet]; [p = 0.003]) (Fig. 3B). In Table 3 we reported the median survival times for each mutated subgroup who presented the most frequent mutation.

**Table 3:** Median survival time in subgroups with the most frequent mutations

Gene mutatation	N° patients	Median survival time (months)	95% Confidence interval
Total-SNV	38	149	60–238
IDH1-SNV	20	194	135–253
EGFR-SNV	8	19	9–28
PIK3CA-SNV	8	194	not evaluable
Total -CNV	35	20	18–22
EGFR-CNV	25	19	17.21
CDK4-CNV	8	19	0–39
Total-Fusion	21	20	17–22
EGFR-fusion	17	19	15–22

*CNV* copy number variants, *SNV* single nucleotide variants

### NGS and molecular tumor board

Among all the patients included in this study, only four cases were discussed by the institutional molecular tumor board of our hospital—a panel of experts who analyze specific cases of cancer patients to identify cutting-edge personalized therapeutic options [35]. One patient with *ERBB2* mutation received no treatment indication for poor clinical performance; one patient with *CDK4* mutation was offered treatment with abemaciclib, a CDK4/6 inhibitor indicated for the therapy of patients with breast cancer [36], but did not start the therapy because of the onset of autoimmune thrombocytopenia. One patient with *PIK3CA* mutation was offered alpelisib, a PI3Kα-inhibitor indicated for the treatment of advanced breast cancer [37], but could not proceed with treatment due to clinical deterioration. Finally, one patient with *RET* fusion was enrolled in a phase 1/2 study with pralsetinib (BLU-667), a highly-selective RET inhibitor currently investigated in the treatment of different solid tumors [38].

## Discussion

Our findings show that most patients with glioma present mutations such as SNVs, CNVs, or gene fusions; however, the prevalence of currently targetable mutations in primary brain tumors remains low. As shown in Table 2, most of the mutations detected were theoretically actionable through either off-label therapies or ongoing clinical trials. Based on guidelines for the clinical interpretation of somatic variants [20], most mutations were classified as TIER II C, meaning that FDA-approved therapies are available for that specific variant but in a different tumor type or there are ongoing trials in this tumor type.

This is true for SNVs for *IDH1, IDH2, EGFR, BRAF, PIK3CA, FGFR3, ERBB2, CNVs for EGFR, PIK3CA, MET, CDK4/6*, and fusion for *EGFR, MET* and *FGFR3*. A single-nucleotide variant of *BRAF* was also classified as Tier IA, meaning that an FDA-approved therapy is available for all types of tumors bearing that variant [20]. Finally, patients with *KRAS* mutation could potentially be referred to a basket trial that is currently investigating the safety and efficacy of the MEK inhibitor trametinib and the BCL2-family inhibitor navitoclax in patients with different forms of advanced or metastatic solid tumors [39].

Despite the theoretical availability of molecularly matched therapeutic options, only four out of 67 patients were referred to a targeted treatment either off-label or as part of a clinical trial. This is because genomic profiling and identification of actionable mutations often occur too late in the diagnostic process, when a targeted approach is often considered unfeasible, given the poor clinical status of the patient. Therefore, the future goal for glioma-targeted therapy is to implement routine and timely genomic profiling, moving toward a more personalized precision medicine strategy in neuro-oncology [9]. Besides identifying cancer-specific mutations, new molecular diagnostic tools are available, such as RNA sequencing, proteomics and epigenetic analysis, which may allow the identification of new targetable mutations [3, 9, 40]. Moreover, the routine integration of molecular markers into the diagnostic work-up may lead to the identification of predictive or prognostic biomarkers that help determine the most appropriate treatment strategy for each patient [3].

Despite the advances in the molecular characterization of gliomas, before personalized medicine becomes a reality for patients with CNS tumors, further efforts are needed to identify effective agents [3]. As of now, the results obtained with targeted therapy are still poor, partly because of the peculiarities of CNS tumors, such as immunosuppression and the presence of the blood–brain barrier, which make most of the recommended targeted therapies otherwise used in oncology ineffective against glioma [41]. Given that classic targets, such as the p53 and retinoblastoma pathway and *EGFR* targeted therapy, have failed to provide clinical benefit in patients with glioma, new potential strategies are currently focusing on immunotherapy, tumor microenvironment, and a combination of several efficacious methods [42].

Another important step toward implementing precision medicine in glioma management is the creation of large brain tumor biobanks, which are essential for advancements in diagnosis, understanding pathogenic mechanisms, and developing patient-derived models [40]. Biobank implementation requires significant infrastructure and institutional resources. At the same time, the role of the surgical oncologist is key to ensuring the quality, preservation, and processing of surgical tissue, which has major downstream effects on translational clinical efforts [40]. Moreover, since one of the main problems is the limited amount of tissue that is usually available for molecular profiling, our neurosurgery performs ‘en block resection’, to avoid tissue fragmentation, provide good quality tissue for the biobank and perform molecular analysis. Innovative and less invasive molecular profiling strategies are under development, which include the exploration of liquid biopsy specimen or the evaluation of cerebrospinal fluid [43, 44].

Lastly, modern trial designs in precision medicine, such as ‘basket and platform trials’, have recently emerged in the glioma trial landscape. ‘Basket trials’ allow the inclusion of patients presenting with a specific driver mutation irrespective of the underlying tumor type, while in ‘platform trials’, patients are screened for a broad range of driver mutations and assigned to a particular treatment based on the results of the genetic testing. These innovative strategies could help overcome the limitations of current clinical trials in glioma, which are constrained by a low number of prospectively treated patients, thus accelerating the development of targeted treatments in patients with primary CNS tumors [45].

## Conclusion

Although limited to a single center, the results of our study are in line with those reported by the TCGA database on a larger population and confirm that most patients with glioma present mutations such as SNVs, CNVs or gene fusions. This finding emphasizes the importance of routine and timely molecular profiling in patients with glioma to favor implementing an individualized diagnostic and treatment approach in this population. Glioma patients presenting with actionable mutations could benefit from the expansion of precision medicine in the neuro-oncology field, thanks to the availability of targeted agents, either off-label or currently in the research and development phase. This aspect could increase the chances of response in a disease still characterized by a poor prognosis.

Future studies are needed to identify better targeted therapy options, as well as to clarify when the targeted approach should be started, either at the initial diagnosis or recurrence, and what is the best timing for molecular profiling with respect to treatment start.

## Data Availability

The data supporting the findings of this study are available upon request to the corresponding authors (Veronica Villani). The data are not publicly available because they contain information that could compromise the privacy of research participants.

## Abbreviations

*BRAF*: B-Raf proto-oncogene, serine/threonine kinase
*CDKN2A*: Cyclin-dependent kinase inhibitor 2A
CNS: Central nervous system
CNV: Copy number variants
*EGFR*: Epidermal growth factor receptor
GBM: Glioblastoma
*IDH*: Isocitrate dehydrogenase
NGS: Next-generation sequencing
OS: Overall survival
PFS: Progression-free survival
*PTEN*: Phosphatase and tensin homolog
SNV: Single nucleotide variants
*TERT*: Telomerase reverse transcriptase
*TP53*: Tumor protein p53
TTP: Time to progression from the NGS analysis
